# The role of two-stage repair in modern hypospadiology

**DOI:** 10.4103/0970-1591.40618

**Published:** 2008

**Authors:** Aivar Bracka

**Affiliations:** Russells Hall Hospital, Dudley, West Midlands, England

**Keywords:** Hypospadias, two-stage repair, grafts, balanitis xerotica obliterans

## Abstract

Hypospadias surgery continues to evolve. The enthusiasm for flap-based urethroplasty is waning and instead there is an increasing preference for urethroplasty that uses either the urethral plate alone or in combination with grafts. From the vast armamentarium of hypospadias repairs that are still in use, the author suggests a simple protocol of just three closely related procedures with which we can now repair almost all hypospadias. The tubularised incised plate (TIP) repair and the ‘Snodgraft’ modification of the TIP principle are simple and effective one-stage solutions when partial circumference urethroplasty is required. Conversely, the Bracka two-stage graft repair remains an ideal and versatile solution when a full circumference urethroplasty is required. It is particularly appropriate for severe primary hypospadias associated with a poor plate and marked chordee and also to replace a scarred, hairy or balanitis xerotica obliterans diseased urethra in re-operative salvage hypospadias.

## INTRODUCTION

Worldwide, the use of staged operations for hypospadias remained the norm in many centres during the decades that followed the introduction of the Denis Browne repair[1] and its subsequent derivatives.

These operations were popular more for their technical simplicity than for the quality of reconstruction that could be achieved.

Chordee correction and meatotomy were undertaken as a preliminary first-stage procedure and if need be this first stage was repeated until a satisfactorily straight penis had been achieved. Only then at a subsequent operation was a buried skin strip urethroplasty performed to leave a meatus either at the corona or attached onto the ventral aspect of the glans.

The gurus of that era regarded an irregular and potentially hair-bearing urethra, ending in a dystopic ventral meatus, to be adequate for both functional and aesthetic purposes. As it was not customary to undertake any long-term follow-up, they may have been genuinely oblivious to the late problems and concerns that these staged ‘ventralising’ repairs created. Perhaps the other reason for accepting relatively crude results was that they simply had nothing better to offer at that time.

Although one-stage ‘terminalising’ repairs were in existence during that era, they were technically challenging and in most hands had such prohibitive complication rates that they were often regarded as more of a ‘surgical ego trip’ than a viable option for hypospadias correction. Indeed children operated in our own establishment with single-stage tubed flap repairs such as the Broadbent and Mustarde techniques during the 1960s and 1970s, were found to have had a more than 90% complication rate at adult review.[2]

Tubularised preputial island flap repairs achieved respectability in the hands of Asopa during the 1970s, but it was not until the 1980s that one-stage repairs gained universal acceptance, largely due to the persuasive advocacy of John Duckett in the United States. It then appeared that hypospadias surgery was at last reaching its evolutionary end-stage, with the prospect of reliably correcting almost any deformity in just one operative intervention and furthermore achieving the goal of a meatus on the apex of the glans. It is not surprising therefore that the old, staged ‘ventralising’ repairs, which required more hospitalisation and achieved a less complete correction, rapidly slipped into obsolescence.

## PROBLEMS WITH 1980S ONE-STAGE REPAIRS

Like the majority of surgeons in the early 1980s, the author was initially seduced by the tide of enthusiasm for this emerging one-stage philosophy. The arguments for single-staged protocols, such as MAGPI/Mathieu/Duckett or Asopa TPIF, were irresistible and in principle, the operations seemed sound and looked convincing in diagrammatic form. In reality, however, it became clear to the author from his own experience that there were unavoidable penalties that had to be accepted for the benefit of one-stage convenience.

One had to master some tricky decision making, with a protocol of completely unrelated operations, each with a limited range of application and associated with different shortcomings. This became more confusing as a stream of modifications and new para-meatal flap procedures joined the potential armamentarium. Despite the plethora of operations from which to choose, the objective of creating a natural glans configuration and vertical slit-like meatus with any consistency remained an insurmountable problem. Speaking with colleagues working in other hypospadias centres confirmed that in reality these were common concerns. Publications of that era tended to show diagrammatic representations perhaps with intra-operative photographs, but there was a worrying lack of photographic evidence to confirm the almost universal claims of satisfactory, good or excellent cosmetic outcomes. All too often in the author's experience the outcome was an unattractive glans with a stenosed, puckered or misshapen orifice and with the TPIF, a poorly supported baggy neo-urethra.

Although the Philadelphia group published remarkably low re-operation rates for their surgery (<5% re-operations for all their hypospadias cases), few others could reproduce such results. It is all too easy to equate re-operation statistics with complication or dissatisfaction rates. The longer is the follow-up, the more problems and concerns that come to light. Given an aggressive early discharge policy, little or no follow-up and perhaps a high threshold for re-intervention, the low re-operation statistics doubtless painted an unduly flattering picture of what was being achieved. This is evidenced by the fact that John Duckett himself eventually stopped advocating his ubiquitous, eponymous repair in favour of less problematic onlay island flaps. However, even the safer onlay flap repair was not without its problems, with fistula rates of up to 20% quoted by recognised experts working in internationally renowned institutions.[3]

During the Duckett era, criticisms about cosmetic shortcomings, in particular concerning poor meatal shape, were dismissed as being insignificant. Although it is widely recognised that children are far more tolerant of sub-optimal results than are adults, in the absence of adequate long-term follow-up data, such dismissals might therefore have been a genuine belief. Perhaps however, these dismissals also reflected that the gurus of the 1980s, just as with their ‘ventralising’ predecessors, simply had nothing better to offer at the time.

The long-term reviews of hypospadias repair show that for adolescents and young adults, genital aesthetics can be just as important as functional considerations. In the author's own study,[2] nearly half of more than 200 young adults reviewed were sufficiently concerned about the quality of their repairs to undergo revisional surgery. Similarly 48% of the adolescents in Mureau's study[4] also said that they would consider further surgery if the penis could be made to look more normal. Mureau found that patient assessment of cosmetic results was notably less favourable than the more optimistic views of their surgeons. This might seem a surprising finding when considering that most hypospadias publications claim subjectively good to excellent cosmetic results for the technique that is being promoted or reported on.

## THE REBIRTH OF TWO-STAGE REPAIR

To try and address the various shortcomings of the Duckett protocol, the author reverted back to a two-stage repair in the mid 1980s and published the results of the first 600 cases in 1995.[5,6] This was not a re-incarnation of the obsolete buried skin strip ‘ventralising’ repairs, but a modern two-stage ‘terminalising’ repair that could produce an even calibre hairless neo-urethra with a natural slit meatus and glans configuration. Not only did the results prove to be more sophisticated than with the then available one-stage methods, but also the surgery was relatively straightforward, reliable and reproducible.[7–12] Furthermore, being uniquely versatile it could be used as a universal repair for almost all hypospadias deformities. For a then trainee general plastic surgeon dealing with a still modest number of hypospadias patients, being able to master one straightforward principle of repair and produce refined results in a broad spectrum of primary and re-operative problems was undoubtedly appealing. Employing one versatile method to a high standard seemed preferable to remaining on a learning curve with a whole armamentarium of disparate procedures.

This article primarily addresses the philosophy and the current role for the Bracka two-stage hypospadias repair. Detailed technical operative details have already been described elsewhere.[5,6,13] In essence, the first stage creates a neo-urethral plate. This involves clefting the glans, enlarging the meatus if required and releasing chordee by transecting the native urethral plate and dissecting away any tethering tissue from the corpora. A free graft, taken ideally from the inner prepuce, is quilted down onto the defect and further immobilised with a tie-over dressing until a blood supply has been established. Then after about 6 months, once the new urethral plate is well vascularised and matured, it is tubed to form a urethra. This second stage is effectively the same as the tubularised incised plate (TIP) repair but without the need for a midline dorsal releasing incision, because the new plate is already of adequate width. The inherent versatility allows for application in challenging salvage situations as well as primary repair, because when inner prepuce is no longer available, then alternative graft donor sites can be used to construct the new urethral plate. Both post-auricular Wolfe grafts (PAWG) and buccal mucosa grafts have been successfully used for this purpose and will be discussed later.

**Figure 1:**
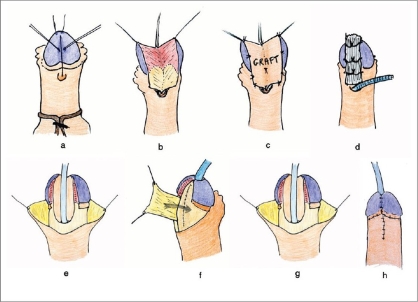
(a-h) The principles of two-stage graft repair in a distal hypospadias

Figure 1 is a diagrammatic representation of the author's repair as originally published in 1995, when it was still being used for primary distal hypospadias.

These are generic diagrams, which can be extrapolated to represent both primary proximal and also salvage hypospadias. Nevertheless, several comments about the operative design are now warranted, given that our knowledge of hypospadias anatomy has continued to evolve over the past decade and we now understand aspects of glans configuration and chordee formation which were less appreciated at the time of original publication.

A common design error in the first stage, for those surgeons still on the learning curve, is to cleft the glans too far posterior. We now appreciate that in most hypospadias deformities there is a variable degree of downward and external rotation of the glans wings. This explains the rather flattened glans, the ventral defect in the foreskin and why the distal extremity of the urethral plate ends on the ventral aspect of the glans. The true apex of the glans is therefore rather more ventral than might be appreciated. If allowance is not made for this and the glans is clefted right back to the apparent rather than the anatomically correct apex, then when the glans wings are derotated and realigned at the second stage, the patient will end up with a somewhat epispadic meatus.

Some may question how from the original descriptions, the first-stage procedure was adequately able to correct chordee, when it did not include degloving of the skin envelope. The first-stage dissection primarily dealt with chordee or tethering that was distal to the meatus. When despite the distal dissection chordee persisted at the level of the meatus or more proximally, further mobilisation was undertaken by undermining the native urethra for a variable distance towards the base of the penis, to allow more extensive dissection and scoring of any tissue that might be restricting the ventral tunica. This sometimes resulted in more straightening, together with further retro-position of the meatus. This manoeuvre perhaps represented an early and less radical implementation of the urethral mobilisation policy that has recently been proposed by Bhat in India.[14]

Formal degloving of the skin envelope was in most cases deferred until the second stage, unless chordee correction was proving to be unduly problematic and it was clear that ventral skin tethering was a contributory factor. Skin degloving at the second stage released any remaining ventral tethering and if despite this degloving there was still any residual chordee on saline erection testing, then at that point a dorsal Nesbit was undertaken before proceeding to the urethroplasty. Deferring full circumference degloving (± Nesbit) until the second stage rather than doing it at the first stage meant that one was dealing with virgin tissue planes on the dorsum of the penis, thereby making it easier to fashion a good dartos ‘water-proofing’ flap to cover the urethroplasty.

The operation as described divides the urethral plate at the outset, thereby making an early commitment to two stages. Often it is very evident that this is required, but with current trends to preserve and utilise the native urethral plate whenever possible, then it is perfectly reasonable to proceed along plate preserving lines in the first instance and only convert to the two-stage option as and when necessary. It, therefore, offers a safety net when the one-stage enthusiast is getting into trouble trying to do a difficult correction. All too often surgeons do become slaves to ‘one-stage machismo’, persevering with ever more extensive dissection of impossibly tenuous flaps, when opting for a reliable and more refined outcome in two stages would have been preferable and safer.

What about timing of surgery? There are two recognised ideal windows of opportunity for hypospadias repair - either during the first year of life or between 3 and 4 years.[7] Most experience with the Bracka repair has been in patients over the age of 3 years. This reflects that the author has worked in a district general hospital rather than a specialised paediatric surgery facility. There is, however, no reason why surgery could not be undertaken in the first year of life where suitable facilities exist, with the proviso that surgery can be completed before the age of 18 months. The period between 18 months and 3 years is a psychologically difficult time for hospitalisation and should be avoided if at all possible.

The author used this two-stage repair as the mainstay of his hypospadias practice for more than a decade and up until the mid 1990s, there was a valid argument for using this method as a convenient and supremely versatile ‘panacea’ repair for all but very minor degrees of primary hypospadias.

Whilst a variety of technically more intricate one-stage flap repairs can produce decent results in the hands of a few gifted individuals, a measure of the worth of this particular method is that it can be mastered by hypospadias surgeons of average ability and by trainees.[8] It can be used safely not only by paediatric surgeons working in specialised children's hospitals,[9,10,13] but also by alternative specialists working in general hospitals[11] and likewise in less sophisticated third world environments.[12]

In these respects the author's two-stage repair proved to be a worthy alternative to the ubiquitous one-stage protocols and it gained increasing acceptance throughout the 1990s. It became initially popular among plastic surgeons, a group who were inherently more comfortable with the concept of using free grafts than were their paediatric surgery colleagues. Over time however, paediatric surgeons have gradually overcome their suspicion of grafts and although today more limited indications remain for a two-stage graft repair, a still increasing number of surgeons are adding this method to their hypospadias armamentarium.

## THE CHANGING ROLE FOR TWO-STAGE REPAIR

### Primary distal

The role of two-stage repair changed dramatically with the advent of the TIP repair. Although Orkiszewski described the TIP concept as a salvage procedure back in 1987,[15] it remained effectively unknown until resurrected by Snodgrass in 1994.[16] Snodgrass began promoting the TIP as an effective method to treat distal primary hypospadias and thus started a revolution in the way we now manage hypospadias.

The TIP repair removed previous objections to one-stage procedures. It is simple, safe and capable of producing cosmetic and functional results that are comparable with the two-stage repair. Hence, it is now the most widely used operation worldwide for routine distal primary hypospadias correction and is indeed my own preferred choice for such cases.

Just occasionally there are still some distal deformities that invite the use of a two-stage repair. The TIP is easy when the glans wings are externally rotated and flattened, with a well-defined groove extending up onto the ventral glans. The truly round or conical glans configuration is uncommon, but when it does occur then it is easier to achieve a truly apical slit meatus by opening up the glans and grafting the resulting cleft to produce a good deep, wide urethral plate extension within the substance of the glans. The urethroplasty at the second stage then becomes very easy. Whilst some technically exceptional hypospadiologists may feel that even in these challenging cases they can produce an anatomically accurate meatus in a single stage, for most surgeons it would be more sensible to create a good meatus using two very simple and safe steps. This is better than struggling to push the TIP repair beyond its natural limits and produce a compromised outcome, which then needs subsequent revision and thereby negates the one-stage advantage.

Occasionally in a distal hypospadias, significant terminal chordee or glans tilt remains despite skin degloving and thorough ventral dissection. Transecting the plate sometimes produces a dramatic improvement. This then necessitates a full circumference urethral reconstruction, something that is achieved with safety and with more refined results using the two-stage graft method rather than a one-stage tubed flap repair.

### Primary proximal

The role of two-stage repair in primary surgery is being further eroded by the increasing use of the TIP for more proximal deformities, although the indications and limitations of the TIP in these more challenging cases are still being explored and determined.

In proximal cases the urethral plate is often poorly defined and when short and tethered it may contribute to the relatively high incidence of chordee that is seen in these more severe forms. If the plate is of reasonable quality and ventral curvature can be corrected without transecting the plate or excessively shortening an already hypoplastic organ with dorsal plications, then an extended TIP may well prove to be a good option. It is probably better than an onlay flap repair, as there is increasing concern being voiced at international meetings that these flap repairs, having poor mechanical support, tend to become patulous with time and patients may then have to contend with problems such as poor ejaculation and post-micturition dribble.

For severe degrees of hypospadias, many surgeons, the author included, still prefer to transect a short, tethered plate and create a longer and wider new urethral plate using an inner prepuce graft as an initial first-stage procedure. This is simple and safe and, should it prove necessary, it leaves scope for further modification of the new plate or any residual chordee, either before or during the second stage. Even in major paediatric centres that used to be totally committed to one-stage flap solutions, this reliable two-stage graft approach is now finding a place.[10]

It is worth noting that it is not necessary to prejudge deployment of the two-stage repair. If in doubt, one can explore urethral plate preserving options in the first instance and convert to two-stage graft repair if progress is unsatisfactory. This argument cannot be said of one-stage tubed flap repairs such as the Koyanagi, which require a commitment from the very outset.

### Re-operative hypospadias

Perhaps the most important and least controversial role for two-stage graft repair today is in re-operative or salvage surgery; indeed, it was in this context that the method was first conceived.

The TIP repair works the best for primary infant hypospadias correction. It is not ideal for re-operative cases where the urethral plate has become scarred or has previously been replaced by grafts or flaps. This applies even more so in adult penises where the defects for re-epithelialisation are much larger and the healing process slower.

**Figure 2:**
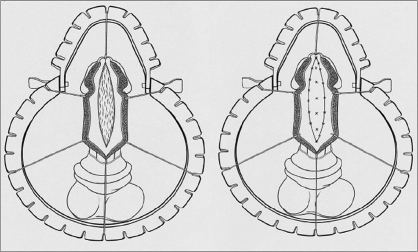
‘Snodgraft’ repair

As in primary surgery, the choice between a one-stage and a two-stage repair is determined by whether the axial integrity of the urethral plate can be maintained. If the plate does not have to be transected and requires only circumference augmentation, then this can be safely achieved with the ‘Snodgraft’ repair. This is a modification of the TIP in which the defect from the dorsal wall releasing incision is closed by quilting in a free graft rather than being left to re-epithelialise spontaneously [Figure 2]. Although the author uses the term ‘Snodgraft’ with permission from Warren Snodgrass, it was Hayes and Malone from the UK who described the use of buccal mucosa graft augmented TIP repairs in 1999.[17]

The ‘Snodgraft’ procedure has reduced the need for two-stage repairs in salvage surgery and may also be useful in some proximal primary repairs where one or other of the mobilised urethral plate strips is of poor quality or doubtful viability and therefore not a good source for outgrowth of new epithelium. Also it is useful in some distal primary cases with a poor glans groove, where it becomes necessary to extend the dorsal midline clefting incision beyond the confines of the urethral plate to allow sufficient advancement of the meatus. Unless that dorsal extension beyond the urethral plate is grafted, it will probably heal back together and lead to meatal stenosis.

**Figure 3:**
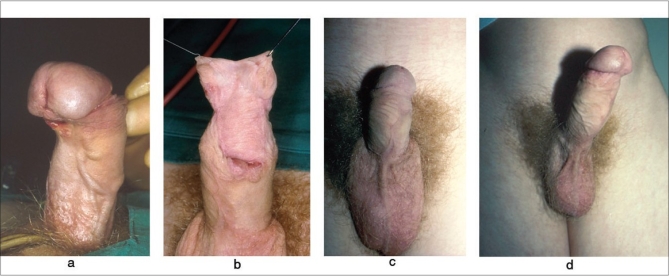
(a-d) Two-stage salvage of failed distal hypospadias repair. (a) shows marked distal chordee, fibrosed glans urethra with coronal fistula, (b) after first stage using residual foreskin hood, (c) healed result and (d) straight erection with full preservation of length plus natural glans and meatus configuration

The indisputable value of the two-stage repair lies in those cases where it is necessary to transect or excise the urethra or urethral plate, thereby creating a full circumference defect. This may be necessary to release severe chordee [Figure 3], to replace a segment of hairy urethra or a urethra that is too scarred and immobile to be amenable to a ‘Snodgraft’ augmentation or to replace a segment of urethra that is diseased with balanitis xerotica obliterans (BXO). Unlike primary repairs, flap options may not even be a consideration in salvage surgery because the patient is already circumcised and has a scarred skin envelope. Even if a flap repair is technically feasible, it may be completely inappropriate because of the presence of BXO, as discussed later.

### Graft options

For post-ischaemic or infective scarring or for hair growth problems, any of the usual three graft donor site options can be used.

1. Inner prepuce is an ideal urethral substitute. It is very thin and flexible, takes reliably, is designed to be moist, has no potential for hair growth and the donor site is both convenient and expendable. It should be used if still available, but if the patient is already fully circumcised then either post-auricular grafts or buccal grafts are both acceptable alternatives.

External prepuce should be avoided if possible as this is not a naturally moist skin and furthermore it does become hairy in some adults.

2. Oral mucosa, whether it is taken from a buccal, labial or lingual donor site, is currently the most widely used alternative to inner prepuce. Although somewhat more capricious with regard to graft take and subsequent behaviour during maturation, it is nevertheless an excellent substitute for urethra, without visible scars or significant donor site morbidity and there is of course no danger of hair growth. Buccal mucosa is bulky and requires aggressive thinning, but generous amounts of mucosa can be harvested from the cheeks, usually sufficient for full circumference replacement of the penile urethra. The cheek donor sites can usually be closed directly for increased patient comfort.

Some have found that graft take and behaviour is more reliable with PAWG than with buccal mucosa;[10,18] however, buccal mucosa offers greater versatility once the learning curve with it has been mastered. Buccal mucosa has therefore displaced PAWGs as second choice donor site in the author's current practice.

Labial mucosa is thinner than cheek mucosa, but limitations on the width that can be comfortably harvested make this better suited to patch urethroplasty, particularly in adults. To avoid lip distortion and occasional sensory problems, these donor sites are more commonly left open to heal by epithelialisation.

Lingual donor sites are still being evaluated.

Buccal and labial donor sites should never be in continuity, because resulting long scar contractures can cause significant oral morbidity.

Problems with graft take can be reduced by adequate thinning of the grafts, extensive use of quilting sutures, longer application of tie-over dressings than when using inner prepuce and appropriate antibiotic prophylaxis against the anaerobic spectrum found in the mouth.

3. PAWGs are full thickness skin grafts harvested from the back of the ear and/or post-auricular sulcus. Such grafts are significantly thicker than inner prepuce but similar to or thinner than buccal mucosa grafts. Like inner prepuce, the post-auricular skin is a flexural skin that is designed to cope with a moist environment and therefore adapts well as a urethral substitute. Although in the author's experience a very short, fine lanugo type of hair growth sometimes becomes apparent in adult patients, coarse hairs are only ever a problem if the donor area strays too far towards the mastoid region. A significant downside with PAWGs is that troublesome donor site keloid scars occasionally occur. More rarely the author has even encountered junctional keloids within the reconstructed urethra, necessitating replacement of the post-auricular skin with buccal mucosa.

All other extra-genital skin graft sites such as groin or inner arm should be avoided altogether. Even when these sites are intrinsically non-hairy, it has been the experience of the author and others that skin from these areas when transferred to a highly vascular glans sponge may subsequently start to grow coarse hairs. It would appear that a change in vascularity can trigger latent hair growth in skin from limb donor sites

### Hypospadias with BXO

The BXO is a descriptive term for male genital lichen sclerosus, a condition of still unknown aetiology. It is nevertheless a very common condition, the author having treated around 1500 patients of whom at least 250 also have hypospadias. It is an important cause of late onset and continuing morbidity after hypospadias repair and accounts for the aetiology in many of the so-called ‘hypospadias cripples’.

In the presence of BXO or a BXO stricture, the therapeutic options for hypospadias repair are very limited.[19]

In the author's extensive experience, meatotomies, dilatations or optical urethrotomies only provide temporary alleviation of symptoms and allow the disease process to continue spreading further proximally.

Augmentation procedures with any form of flap or graft will also fail in the long-term because the disease has not been removed.

Full circumference substitution with any form of skin, whether as flaps or as grafts, will also ultimately fail. New urethras made from extra-genital skin sometimes take from 5 to 10 years before they restricture.

**Figure 4:**
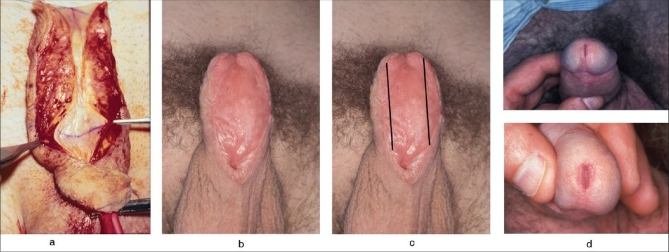
(a) Two-stage buccal mucosa substitution of BXO diseased penile urethra. BXO stricture opened, (b) good graft using both cheeks, 6 months post-op, (c) a 2.5-cm wide strip used for urethroplasty and excess graft discarded and (d) post-operative result showing excellent glans and meatus configuration

Currently, it is the author's view that the only effective solution for BXO is to replace the entire diseased segment of urethra with healthy mucosa. Where the disease is still confined to the penile urethra, this can be achieved with a two-stage buccal mucosal graft substitution [Figure 4]. The author has now salvaged several hundred BXO urethras in this fashion over the past 15 years, so far without problems of disease recurrence. In long neglected disease, usually in adults, urethral strictures can extend back as far as the external sphincter and therefore present a particularly difficult reconstructive challenge. In such cases when there is insufficient buccal mucosa available for reconstruction, it has been necessary to combine buccal mucosa with bladder mucosa to be able to replace the bulbar as well as the penile urothelium.

Buccal mucosa, whilst being a naturally moist mucosa, does fortunately cope with exposure to the air (unlike bladder mucosa) and can therefore be used in a two-stage fashion.[20] This is just as well because large buccal grafts do not behave quite as predictably as preputial or post-auricular skin during the healing phase.[18] Many of the healed grafts retain their original dimensions and stay smooth and moist - even if left untubed for several years. Others undergo a varying degree of post-operative contraction, oedema or keratinisation in the initial months. This may be permanent but is often temporary and with graft maturation and the tissue expanding effect of erections, the graft may recover most or all of its original dimensions within 6 months. It is, therefore, wise to place grafts of wider than required dimensions at the first stage to allow for some possible contraction. If the matured graft remains wider than required at 6 months, it is easy enough to discard the lateral surplus. Conversely, if there is an excessively narrow area, this can be augmented with more buccal mucosa along ‘Snodgraft’ lines when doing the second-stage tubing. What should not be done is to make up the width deficiency by including adjacent skin with the mucosa. Inclusion of skin into the neo-urethra is likely to lead to disease recurrence.

These facts help to explain why there is such a high morbidity rate for one-stage tubed buccal graft repairs in the American experience. In a personal communication, Snodgrass reported 50% re-operation rates for one-stage tubed buccal mucosa grafts when polling hypospadias surgeons at a national paediatric surgery meeting. Complication rates up to 69% have been reported in the literature for one-stage buccal repair of complex hypospadias.[21–23]

In the one-stage version, the new urethra is fashioned from a tubed graft that still has to acquire a blood supply and undergo the maturation stages. Judging the dimensions of the new urethra and meatus is therefore inevitably a ‘guesstimate’. Furthermore, the healing anastamotic junctions tend to contract; therefore, regular calibration of the repair may be required during the first 6 months or so.

It has long been the author's subjective impression that two-stage buccal graft repair is an inherently much safer and more versatile method of full circumference urethral substitution and this view is now gaining increasing support, even in the United States.[24] Preliminary results of an audit of around 300 of the author's two-stage buccal mucosa urethroplasties, still awaiting completion and submission for publication, supports the impression that the re-operation rate after two-stage buccal repair is only a little higher than after two-stage skin substitution (once the surgeon has become used to working with buccal mucosa). This is because the new urethra is made not from a fresh graft that has yet to pick up a blood supply, but from well-vascularised mature tissue that is already a part of the penis. The dimensions of the new urethra and meatus can therefore be planned with accuracy because the healed graft has already stabilised by this stage.

The Duckett legacy has left the North American continent with an enduring commitment to one-stage solutions for all hypospadias surgery. In recent years however, views about two-stage repair, particularly for these challenging situations, have mellowed and favourable reports are starting to emerge.[24]

## CONCLUSIONS

Two-stage graft repair is now largely confined to those situations where it is impractical to maintain the axial integrity of the urethral plate and therefore a full circumference urethral reconstruction becomes necessary.

When the lengthways integrity of the urethra or urethral plate can be maintained, so that only a partial circumference augmentation is required, repair can be safely and effectively achieved in a single stage with either the TIP or the ‘Snodgraft’ modification of the TIP.

Thus it is possible to correct almost all primary and re-operative hypospadias using just three repairs - the TIP, the ‘Snodgraft’ and the Bracka two-stage.

A further advantage of using this simple ‘flap-free’ protocol is that these methods form a logical progression and are technically very closely related. Thus once the two-stage graft repair has been mastered, it is just as easy to deploy the other two repairs. The TIP repair is essentially the same as the Bracka second stage with just the addition of a dorsal releasing incision in the urethral plate. Similarly, quilting a free graft into the dorsal defect of the ‘Snodgraft’ repair will not present any new challenge once the Bracka first stage has been mastered.

The old saying that “there is more than one way to skin a cat” is very true of hypospadias surgery. This proposed protocol and indeed any alternative protocol, will not appeal to everyone and there will be surgeons who have a large experience with flap repairs and remain happy with their results. The highly experienced and technically gifted super-specialist may feel that he can correct almost all of his cases using a diverse variety of single stage procedures and will use the Bracka approach quite sparingly. At the other end of the scale, the generalist for whom hypospadias may be a small proportion of his practice and has to work in rudimentary facilities with crude instruments and sutures may wish to keep things as simple as possible and use the versatile two-stage method for all but straightforward primary distal hypospadias.

In conclusion, whilst indications for the author's two-stage graft repair have reduced over the last decade, they can be applied flexibly to take account of the differing skills, experience and circumstances of the individual surgeon. Two-stage repair will continue to have a role in modern hypospadiology, certainly for the foreseeable future.

## References

[1] Browne D (1949). An operation for hypospadias. Proc R Soc Med.

[2] Bracka A (1989). A long-term view of hypospadias. Br J Plast Surg.

[3] Mouriquand PD, Persad R, Sharma S (1995). Hypospadias repair: Current principles and procedures. Br J Urol.

[4] Mureau MA, Slijper FM, Nijman RJ, van der Meulen JC, Verhulst FC, Slob AK (1995). Psychosexual adjustment of children and adolescents after different types of hypospadias surgery: A norm-related study. J Urol.

[5] Bracka A (1995). A versatile two-stage hypospadias repair. Br J Plast Surg.

[6] Bracka A (1995). Hypospadias repair: The two-stage alternative. Br J Urol.

[7] Manzoni G, Bracka A, Palminteri E, Marrocco G (2004). Hypospadias surgery: When, what and by whom. BJU Int.

[8] Titley OG, Bracka A (1998). A 5-year audit of trainees experience and outcomes with two-stage hypospadias surgery. Br J Plast Surg.

[9] Ferro F, Zaccara A, Spagnoli A, Lucchetti MC, Capitanucci ML, Villa M (2002). Skin graft for 2-stage treatment of severe hypospadias: Back to the future?. J Urol.

[10] Johal NS, Nitkunan T, O'Malley K, Cuckow PM (2006). The 2 stage repair for severe primary hypospadias. Eur Urol.

[11] Price RD, Lambe GF, Jones RP (2003). Two-stage hypospadias repair: Audit in a district general hospital. Br J Plast Surg.

[12] Obaidullah MA (2005). Ten-year review of hypospadias surgery from a single centre. Br J Plast Surg.

[13] Samuel M, Duffy G (2004). Two-Stage urethroplasty Chapter 23 of Hadidi and Azmy's. Hypospadias surgery. An illustrated guide.

[14] Bhat A (2007). Extended urethral mobilization in Incised Plate Urethroplasty for severe hypospadias: A variation in technique to improve chordee correction. J Urol.

[15] Orkiszewski M (1987). Urethral reconstruction in skin deficit. Polish Urol.

[16] Snodgrass W (1994). Tubularized incised plate urethroplasty for distal hypospadias. J Urol.

[17] Hayes MC, Malone PS (1999). The use of a dorsal buccal mucosal graft with urethral plate incision (Snodgrass) for hypospadias salvage. BJU Int.

[18] Tahmeedullah, Khan A, Obaidullah MA (2003). Comparison of prepucial skin, postauricular skin and buccal mucosal graft results in hypospadias repair. J Coll Physicians Surg Pak.

[19] Depasquale I, Park AJ, Bracka A (2000). The treatment of balanitis xerotica obliterans. BJU Int.

[20] Mokhless IA, Kader MA, Fahmy N, Youssef M (2007). The multistage use of buccal mucosa grafts for complex hypospadias: Histological changes. J Urol.

[21] Snodgrass WT, Lorenzo A (2002). Tubularised incised plate urethroplasty for hypospadias reperation. BJU Int.

[22] Mouriquand PD, Mure PY (2004). Current concepts in hypospadiology. BJU Int.

[23] Irani D, Hekmati P, Amin-Sharifi A (2005). Results of buccal mucosa graft urethroplasty in complex hypospadias. Urol J.

[24] Snodgrass W, Elmore J (2004). Initial experience with staged buccal graft (Bracka) hypospadias re-operations. J Urol.

